# Diversity and Ecology of Thrips (Thysanoptera, Insecta) Assemblages in Słowiński National Park—A Biosphere Reserve on the Baltic Coast (Northern Poland)

**DOI:** 10.3390/insects17010119

**Published:** 2026-01-21

**Authors:** Halina Kucharczyk, Marek Kucharczyk, Irena Zawirska

**Affiliations:** Department of Zoology and Nature Protection, Faculty of Biology and Biotechnology, Institute of Biological Sciences, Maria Curie-Sklodowska University, 19 Akademicka Str., 20-033 Lublin, Poland

**Keywords:** biodiversity, species richness, ecological characteristics, host plants, plant communities, environmental factors

## Abstract

Słowiński National Park is one of the 23 national parks in Poland and one of the two situated on the Baltic Coast in the country. It was established in 1967 to protect the most valuable ecosystems: coastal lakes, marshes, peat bogs, meadows, forests, and, above all, the dune belt of the Łebska Spit with its unique moving dunes. We aimed to 1. determine the species diversity and structure of thrips assemblages in the most important biotopes of the Park; 2. determine the geographical distribution and food preferences of thrips species; and 3. determine which environmental factors influence the diversity of insect assemblages and which thrips species distinguish these assemblages. The method used in the quantitative research was based on the use of a scoop method; it was supplemented by qualitative research (shaking branches of trees and searching for insects on their host plants). The studies were carried out in 1991 and 1999–2001 in fourteen plant associations. A total of 90 thrips species (nearly 40% of the Polish fauna) were recorded, including 71 in quantitative and 74 in qualitative samples. The study also revealed a significant correlation between the thrips assemblage composition and the following environmental factors: soil moisture, light intensity, general nutrient availability, and soil salinity. In addition, the thrips species with the most significant impact on assemblage composition were identified. The relatively high number of species found, including *Taeniothrips zurstrassenii* Zawirska, a species new to science, and others rarely recorded in Poland, highlights the value of the SNP habitat diversity in maintaining high Thysanoptera diversity.

## Simple Summary

Słowiński National Park is situated on the Baltic Coast in Poland. It was established in 1967 to protect the most valuable ecosystems, including coastal lakes, marshes, peat bogs, meadows, forests, and, above all, the unique moving dunes of Europe. We aimed to determine the thrips species composition and diversity in the different land ecosystems of the Park. The studies were carried out in 1991 and 1999–2002 across 14 plant communities. We used both quantitative and qualitative methods in our research. A total of 90 thrips species (nearly 40% of the Polish fauna) were recorded, including 71 in quantitative and 74 in qualitative samples. The species-richest habitats were the *Leucobrio-Pinetum* forest and meadows classified as *Arrhenatheretalia elatioris*, with 36 and 33 species, respectively. In contrast, the poorest habitats were the peat bog communities *Caricetum gracilis* and *Carici canescentis-Agrostietum caninae* with 10 species, and *Erico-Sphagnetum medii* (17 species). The study also revealed a significant correlation between the thrips assemblage composition and environmental factors, i.e., soil moisture, light intensity, general nutrient availability, and soil salinity.

## 1. Introduction

National parks cover the most valuable floristic and faunistic areas in the world. In Poland, 23 national parks exist; Słowiński National Park (SNP) is one of the two national parks (the Woliński NP is the other) situated along the southern Baltic Coast in the country. The SNP covers 32,744.03 hectares, including 21,572.89 hectares of land and 11,171.14 hectares of the Baltic Sea’s coastal waters (Latitude: 54.697–Longitude: 17.359) [1]. According to the geobotanical regionalization, the SNP is situated within two regions: the Baltic Coast and the South Baltic Coast and is located in the Pomeranian Divide, the Central-European Province, the area of European Deciduous and Mixed Forests, the Holarctic [2].

The Park was established in 1967 to protect the most valuable and endangered ecosystems, including coastal lakes, marshes, peat bogs, meadows, forests, and above all, the dune belt of the Łebska Spit, which features Europe’s unique mobile dunes that can reach a height of over 30 m above sea level. The Park’s exceptional natural values, diversity, and unique ecosystems led to its designation as a Biosphere Reserve in 1977 and its inclusion in the UNESCO “Man and the Biosphere” (MAB) program. In 1995, it was included in the Ramsar Convention list of Wetlands of International Importance. The land areas of the SNP are the core of the “Ostoja Słowińska” PLH220023 and “Pobrzeże Słowińskie” PLB220003 sites, which are elements of the European Union Natura 2000 network. Although not particularly plant species-rich, nearly 80% of its vegetation is native. The species found here are typical of a maritime climate—cool and windy, with a short growing season—and have low trophic requirements, thriving in impoverished soils. In addition to native plants, several species native to boreal, Atlantic, and oceanic climates can also be found here [1,2,3,4].

A characteristic feature of Słowiński National Park’s ecosystems is their great diversity and enormous dynamics, expressed in the constant changes occurring in both their inanimate and animate components. The Park’s most remarkable peculiarity is the mobile dunes, which slowly (about 10 m per year) but continuously shift eastward—following the prevailing winds—in a process changing their shapes and destroying plant communities in their path. Once established, plant communities often undergo further changes, transforming into other ecosystems. For example, in the deflation depressions in the grey coastal dunes, peat bogs form or become overgrown with pine forests, while raised bogs transform into boggy forests [5,6]. High air and soil humidity in the SNP means that many species considered forest species in the interior of the country also thrive here in open plant communities without the shelter of trees. Species reaching the limits of their geographical range are common and often numerous on the Łebska Spit. These include boreal and Arctic species typical of the oceanic and Atlantic climate: *Erica tetralix* L., *Carex arenaria* L., *Myrica gale* L., and *Rubus chamaemorus* L. as well as species considered to be glacial relicts: *Calamagrostis stricta* (Timm), *Ledum palustre* L., and *Empetrum nigrum* L. [1,3,7].

The SNP’s land area comprises two distinct geomorphological units, each differing in terrain, subsurface character, and water conditions. The first, located in the Baltic Coast Region, is the Łebska Spit, composed of deep dune sands. It stretches along the sea coast in a narrow strip (up to 9 km wide) from the mouth of the Łupawa River in the west to the mouth of the Łeba River in the east. The second unit, the flat, peaty, and marshy Gardno-Łebsko Lowland, lies between Lakes Łebsko and Gardno. Its southern boundary is marked by a series of post-glacial terminal moraines, located on the Moraine Plateau, which is significantly higher above sea level than the Gardno-Łebsko Lowland. The highest moraine elevation is Rowokół Hill (115 m above sea level), located within the SNP.

The unforested coastal dunes of the Łeba Spit are covered by psammophilous grasses and individual trees. In the final stage of succession, they are overgrown by coastal coniferous forest, where soil-forming processes lead to the formation of gley podzol. Dune and forest plant communities occupy the largest area on the spit, while peat bog and reedbed communities are less common and occur on a much smaller scale. The Gardno-Łebsko Lowland is predominantly a plain, slightly elevated above the water level of both lakes, with a persistently high groundwater table that supports the development of peat-forming vegetation. Within the Lowland, as well as on the moraine hills, there are large forest complexes on podzolic or podzolic-mucky soils. The most common plant communities in the Lowland include lowland peat bogs, poor acidic meadows, reed beds, and forest communities [4,7].

The state of knowledge about the entomofauna of Polish national parks is very diverse and generally insufficient, especially in the two Parks on the Baltic coast [8]. In Słowiński National Park, the only orders whose occurrence has been relatively well documented are Heteroptera [9] and Odonata [10,11]. Among other orders, single species of beetles, butterflies, and flies have been recorded [3,8]. Data on the thrips fauna in the Baltic Sea region are also scarce. The exceptions include observations of Thysanoptera in the Curonian Spit and the Kaliningrad Region [12,13], as well as on the northern Baltic Sea coast in the Scandinavian countries [14,15,16,17].

Worldwide research on thrips faunas in the most valuable natural areas, such as reserves and national parks, is typically limited to diversity studies, checklist preparation, descriptions of dietary preferences, and comparisons with regional faunas. The relationships between thrips assemblages occurring in diverse plant communities are less frequently studied [18,19,20,21]. Of the 23 national parks in Poland, such relationships have so far been studied in Roztocze National Park [22,23], Poleski National Park [24], Ojców National Park [25], Kampinoski National Park [26], and Babia Góra National Park [27].

The order Thysanoptera comprises small, slender insects with a relatively inconspicuous appearance. About 6400 species of thrips are known worldwide, distributed across two suborders, Terebrantia and Tubulifera, and about 220 occur in Poland [28,29]. These insects have discreet body coloration, often yellow, brown, or black, and are characterized by narrow wings fringed with fine hairs. The body length of most European species ranges from 1 to 5 mm, with an average of 1.5–2.5 mm. The shorter lengths are characteristic of the Terebrantia, whereas the highest values are exhibited by Tubulifera suborder species. Larvae are morphologically similar to adults but are wingless and whitish, yellowish, or reddish. Thrips species exhibit considerable heterogeneity in their nutritional and habitat requirements. Among terebrantians, phytophagous species associated with herbaceous plants predominate, while fungivorous species living under the bark of dead trees predominate among tubuliferan thrips. Their mouthparts are most often used for piercing and sucking the contents of plant or fungal cells [30]. Most thrips species are characterized by the ability to move freely, particularly during seasonal migrations and cryptic behavior [30,31]. These organisms have been observed to stop on various plants while feeding, often at random. As a result, one plant species may contain both species related to it in terms of nutrition and development, as well as completely alien species, both in natural plant communities and in cultivated monocultures, where they may cause significant economic losses [32,33,34]. The biology of thrips, including life-cycle length, overwintering sites and wintered stages, migration ability, and body polymorphism (body colour, wing form), is linked to environmental factors such as temperature, light, humidity, and solar radiation [30,35,36,37,38].

Due to the lack of data on the thrips fauna in the Słowiński National Park, the aim of this study was to: 1. determine the species diversity and structure of Thysanoptera assemblages in the most important biotopes of the Park; 2. determine the geographical distribution and food preferences of thrips species; 3. determine the relationships between thrips species and their hosts; and 4. determine which environmental factors influence the diversity of insect assemblages and which thrips species distinguish these assemblages.

## 2. Materials and Methods

### 2.1. Study Area and Study Sites

**Figure 1:**
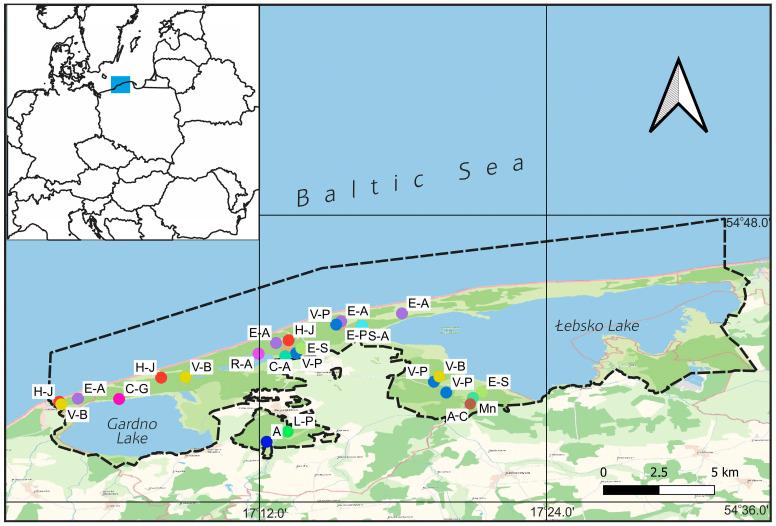
Słowiński National Park—study area and study sites. The dashed line marks the boundaries of the SNP. Acronyms of plant communities: L-P—*Leucobryo-Pinetum*, E-P—*Empetro nigri-Pinetum*, E-A—*Elymo-Ammophiletum*, H-J—*Helichryso-Jasionetum*, V-P—*Vaccinio uliginosi-Pinetum*, S-A—*Sphagno squarrosi-Alnetum*, R-A—*Ribeso nigri–Alnetum*, V-B—*Vaccinio uliginosi-Betuletum pubescentis*, C-G—*Caricetum gracilis*, C-A—*Carici canescentis-Agrostietum caninae*, E-S—*Erico-Sphagnetum medii*, Mn—*Molinietalia caeruleae*, A-C*—Angelico-Cirsietum*, A—*Arrhenatheretalia elatioris*.

The study was conducted across both distinct geomorphological units within the SNP’s land area. The first, located in the Baltic Coast Region, is the Łebska Spit, composed of deep dune sands. The second unit, the flat, peaty, and marshy Gardno-Łebsko Lowland, lies between Lakes Łebsko and Gardno. Materials were collected in all SNP Protection Districts (Rąbka, Smołdziński Las, Rowy, Kluki, and Smołdzino), except for the Żarnów District, located in the northeastern part of the Park. For the research, we selected 14 plant communities that are the most characteristic of the SNP. A total of 24 sites were explored (Figure 1, Table 1, with the acronyms of the plant communities presented in parentheses).

I. Coastal dunes—raised and in deflation depressions—*Elymo-Ammophiletum* (E-A) and *Helichryso-Jasionetum* (H-J)

White and gray coastal dunes stretch along the entire coast of the SNP. The white dunes, formed into ridges, mounds, or dune hills, are bare or partially covered with psammophilous plants of the *Elymo-Ammophiletum* association. It is characterized by the absolute dominance of *Calamagrostis arenaria* (L.) Roth. In more sheltered locations, *Corynephorus canescens* L. and *Carex arenaria* L. also occur, and among the sparse dicotyledons are *Hieracium umbellatum* L., *Viola tricolor* L. subsp. *curtisii* (E. Forster) Syme, and, in some places, *Salix repens* L. subsp. *repens*. Different habitat conditions prevail on the coastal gray dunes in established deflation depressions. Here, a relatively floristically rich psammophilous grassland community, *Helichryso-Jasionietum*, develops despite the poor soil conditions. Common species include *Carex canescens* L., *C. arenaria*, *Calamagrostis arenaria*, and *Festuca villosa* Schweig in some locations. The most common dicotyledonous plants include *Jasione montana* L., *Hieracium umbellatum*, *Hypochoeris radicata* L., *Artemisia campestris* L., *Pinus sylvestris* L. and occasionally *Helichrysum arenarium* (L.) Moench and *Calluna vulgaris* (L.) Hull. Less frequently, *Salix repens*, *Lathyrus japonicus* Willd. subsp. *maritimus*, and *Epipactis atrorubens* (Hoffm.) Besser occur [1,39].

Sample collections sites: E-A on the dunes in Rowy, Czołpińskie Dunes (forest section 19) and in Boleniec; H-J—on dunes in Rowy (f.s. 33), along a red tourist route (f.s. 22), on Dołgie Małe Lake (f.s. 29).

II. Forest communities: fresh pine forests—*Leucobryo-Pinetum* (L-P); bog coniferous forest—*Empetro nigri-Pinetum* (E-P), *Vaccinio uliginosi-Pinetum* (V-P); wet birch forests—*Vaccinio uliginosi-Betuletum pubescentis* (V-B), and alder forests—*Sphagno squarrosi-Alnetum* (S-A), *Ribeso nigri-Alnetum* (R-A).

Within forest communities, the most common biotopes are fresh and moist pine forests, including coastal crowberry forests. Boggy deciduous forests, primarily birch and alder, are common but occupy smaller areas. Pine forests are composed of *Pinus sylvestris* L., *Betula pendula* Roth., *Betula pubescens* Ehrh., *Picea abies* (L.) H. Karst, and rarely *Quercus robur* L. and *Fagus sylvatica* L. In wetlands, the dominant tree is *Alnus glutinosa* (L.) Gaertn. The composition of the forest floor depends on the soil and water conditions within each forest community. The rarest and most valuable are the mostly relic beech and beech-oak forests. Also interesting are the riparian forests, which occupy a negligible area, due to their distinctiveness from the other forest communities in the SNP. Due to their small area, no quantitative samples were taken in the last two forest types [1,39].

Sample collections sites: L-P—along the nature trail to the top of Rowokół (f.s. 124); E-P—along a red tourist route on Czołpińskie Dunes (f.s. 19, 23); V-P—in Czołpiński forest (f.s. 19), in Kluki forest (f.s. 75, 76) and in Smołdziński forest (f.s. 23); V-B—Kluki (f.s. 74), on Dołgie Małe Lake (f.s. 29), on Gardno Lake (f.s. 35); S-A—along route between Łebsko Lake and dunes on Baltic Sea (f.s. 17); R-A—on Dolgie Wielkie Lake (f.s. 24).

III. Raised and transitional peat bogs and fens—*Caricetum gracilis* (C-G), *Carici canescentis-Agrostietum caninae* (C-A), *Erico-Sphagnetum medii* (E-S).

Sedge meadow (*Caricetum gracilis*) is a widespread eutrophic community. It occurs in river valleys, shallow lake basins, stream banks, and small water bodies. The peat bog is clearly dominated by *Carex gracilis* Curtis, with other abundant species including *Galium palustre* L., *Iris pseudacorus* L., *Equisetum fluviatile* L., *Poa palustris* L., *Glyceria maxima* (Hartm.) Holmb., *Phalaris arundinacea* L., and *Menyanthes trifoliata* L. *Carici canescentis -Agrostietum caninae* occurs in permanently wet areas with acidic soils, typically occupying such sites as peaty depressions within meadows. Specific species of these acidic fens include *Agrostis canina* L, *Carex canescens* L., *Carex echinata* Murray, *Viola palustris* L., *Juncus filiformis* L., and *Veronica scutellata* L. *Erico-Sphagnetum medii* is a widespread association in the West-Central European lowlands located in the Atlantic climate zone. Characteristic species include *Erica tetralix* L., *Andromeda polifolia* L, *Oxycoccus palustris* Pers, *Eriophorum vaginatum* L., and peat mosses (genus *Sphagnum*) [1,39].

Sample collections sites: C-G and C-A—in Rowy Protection District (f.s. 32) and on Dołgie Wielki Lake (f.s. 23); E-S—on Ciemińskie Bogs near Łebsko Lake (f.s. 65) and on Dołgie Wielkie Lake (f.s. 23).

IV. Moist and fresh tall-herb meadows: *Molinietalia ceruleae* (Mc), *Angelico-Cirsietum* (A-C), and *Arrhenatheretalia elatioris* (A).

Moist meadows of the order *Molinietalia caeruleae* are permanently or seasonally moist communities with a high proportion of grasses, including *Molinia caerulea* (L.) Moench, *Deschampsia cespitosa* (L.) P. Beauv., and *Holcus lanatus* L. and a highly diverse range of dicotyledonous plant species. The *Angelico-Cirsietum oleracei* association, dominated by *Cirsium oleraceum* (L.) Scop., is particularly noteworthy. It is also accompanied by *Polygonum bistorta* L., *Cirsium rivulare* (Jacq.) All., *Rumex acetosa* L., and *Lathyrus palustris* L. At the station designated in the research as Mn, the following species were found: *Ranunculus acris* L., *Galium palustre* L., *Sanguisorba officinalis* L., and *Rhinanthus serotinus* (Schönh) Oborny. Fresh meadows belonging to the order *Arrhenatheretalia elatioris* are rich in species communities occurring on fresh mineral soils with alkaline or slightly acidic pH. Characteristic plants of these meadows include *Arrhenatherum elatius* (L.) P. Beauv. ex J. & C. Presl, *Dactylis glomerata* L., *Bromus mollis* L., *Daucus carota* L., *Heracleum sphondylium* L., *Pastinaca sativa* L., *Pimpinella major* (L.) Huds., *Trifolium pretense* L., and *Lotus corniculatus* L. [1,39,40].

Sample collections sites: Mc, A-C—near Kluki Stare (f.s. 65); A—on the Łupawa river.

### 2.2. Field and Laboratory Procedures

The material was collected in 1991 (12–19 June, 15–24 August)—qualitative samples only and more intensively in 1999–2001 (1999—16–30 August; 2000—19–29 May, 19–31 July, 1–12 September; 2001—11–27 May, 25 June–8 July, 26 July–12 August, 26 August–6 September, both qualitative and quantitative samples).

Samples were collected in floristically uniform patches of SNP plant communities. The number of sites in a given community ranged from two to three. The study focused on collecting both quantitative and qualitative samples from the same or nearby sites and, in the case of the latter, from similar plant communities characteristic of the SNP (Figure 1).

Quantitative samples were collected using a scoop method, with each sample consisting of 100 scoop strokes. Qualitative samples were collected throughout the growing season from the most abundant plants at both research sites and other areas of the SNP. Qualitative samples allow identification of species whose entire life cycle or developmental stages take place in hidden areas of the plant, such as leaf sheaths, between the glumes in grass inflorescences, or flower and leaf buds. For this purpose, whole plants of one species or parts thereof were collected, placed in cloth bags, and analyzed in the laboratory. In the case of protected plants and when sampling trees and shrubs, the method of shaking flowering or leafy branches, shoots, or whole plants was used. Dead branches and trunks lying on the forest floor were also shaken. From small areas densely covered with a single species, such as lily of the valley, broad bean, or common heather, scoop samples were collected with fewer strokes and treated as qualitative samples.

Thrips specimens collected in quantitative and qualitative samples were placed in tubes containing AGA solution (10 parts of 60% ethyl alcohol, 1 part of glycerin, and 1 part of acetic acid). Due to the small size of the insects, microscopic slides of adults were prepared for identification according to the method described by Mound and Kibby [41]. After clearing in lactic acid, larvae were mounted in Berlese’s solution [42]. Adults were identified using keys developed by zur Strassen [43] and Schliephake and Klimt [44], and larvae were identified using the key by Priesner [45] and Vierbergen et al. [46].

All materials are deposited in the Thysanoptera collection of the Department of Zoology and Nature Protection of Maria Curie-Skłodowska University in Lublin (Poland).

### 2.3. Characteristics of Thrips Species Recorded in the SNP

By analyzing the results from multi-species studies of Thysanoptera assemblages in the selected SNP plant communities, species dominance was calculated [47]. Only adult Thysanoptera individuals collected during quantitative studies (1999–2001) were included in the dominance calculations. Individual dominance is the percentage of individuals of a given species among the total number collected in a given phytocoenosis. Five dominance classes were distinguished: eudominants—constituting 20% or more of all individuals; dominants—from 10.0% to 19.9%; subdominants—from 5.0% to 9.9%; recedents—from 1.0% to 4.9%; and subrecedents—constituting less than 1.0% of individuals (Table 1).

**Figure 2:**
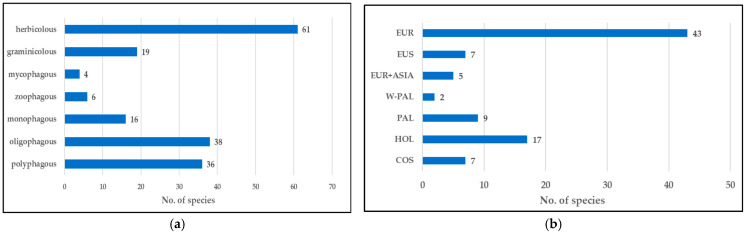
Characteristics of feeding preferences (**a**) and geographic range (**b**) of thrips species collected in Słowiński National Park. Abbreviations: COS—cosmopolite, EUR—European, EUR+ASIA—European and Asian, EUS—Eurosiberian, HOL—Holarctic, PAL—Palearctic, W-PAL—West-Palearctic.

The information contained in the aforementioned keys, together with the article by Zvarikova et al. [48] and our own observations, served as a basis for characterizing all recorded thrips species, taking into account their food preferences, habitat, and geographical range (Figure 2, Supplementary Materials Table S1). Names of plants and plant communities were verified according to Flora Polski [49] (Table S1).

### 2.4. Data Analysis

To investigate environmental effects on the diversity of the thrips assemblages, we considered six explanatory variables based on indicator values of Ellenberg’s ecological scales: light intensity (L), temperature (T), soil moisture (F), soil reaction (R), general nutrient availability (N), and soil salinity (S). Mean characteristic indicator values were calculated for all sample plots based on the cover/abundance values using a 10-degree scale of plant species in the phytocoenosis [50].

The following ecological indices were used to analyze the thrips fauna: species richness (SP), the Shannon–Wiener diversity index (S-W), the Simpson dominance index (1-D), and Fisher’s alpha (FISH). The Jaccard and Bray–Curtis formulas were used to calculate faunistic similarities among habitat types [51].

Non-metric multidimensional scaling (NMDS) ordination analysis in the PAST 5.3 package was used to identify similarities in the dataset and to detect whether any potential environmental gradient influenced the thrips fauna. We used the raw species-abundance data from each study site and the Sørensen (Bray–Curtis) distance. Additionally, canonical correspondence analyses (CCAs) with the forward selection (FS) procedure (999 test permutations) were used to identify the most critical environmental drivers for thrips assemblages [52].

Using the similarity percentage method (SIMPER) [50], we identified the species responsible for the faunistic dissimilarity (Bray–Curtis matrix) among the four main habitats: coastal dunes and forests (E-A, H-J, L-P, E-P), bog forests (V-P, S-A, R-A, V-B), peat bogs and fens (C-G, C-A, E-S), and meadows (Mn, A-C, A).

We also performed indicator species analysis using IndVal, which identified indicator species for each site type. In this analysis, statistical significance (*p* < 0.05) was estimated by 9999 random permutations of sites across groups. CCAs were performed in Canoco 5.0 [53], and NMDS, Simper, and IndVal were performed in the PAST 5.3 program [50].

## 3. Results

### 3.1. Characteristics of Thrips Species and Diversity of Thrips Assemblages

A total of 22,059 specimens (16,964 adults and 5095 larvae) of thrips (Thysanoptera), classified into 90 species, were collected during the research conducted in 14 characteristic plant communities in Słowiński National Park in 1991 and 1999–2001. Most of them (80%) belonged to the suborder Terebrantia and two families—Aeolothripidae (6 spp.) and Thripidae (66 spp.). The second suborder, Tubulifera, was represented by a single family, Phlaeothripidae, comprising 18 species. In the quantitative and qualitative studies, we found a similar number of species: 71 and 74 taxa, respectively. Eighteen species were found exclusively in samples collected with an entomological net in the quantitative study, and 19 species were solely collected in qualitative samples obtained by shaking plants, tree branches, or tapping dead trunks lying on the forest floor at or near the study sites (Supplementary Materials Table S1).

Noteworthy are the significant differences in the number of species collected across the various plant communities. The highest number of species was found in the coniferous forests growing on dunes (L-P, 36 spp.), while slightly fewer species were collected in the fertile, plant species-rich fresh meadows (A, 33 spp.). In contrast, the lowest diversity was exhibited by the peat bogs and fens, where 10 species were recorded in each. In the other insect assemblages, from 14 (S-A) to 24 (E-P and A-C) species were recorded. Excluding the eudominant and dominant species, only single specimens of most of the recorded quantitative study taxa were captured (Table 1).

Only two graminicolous species, *Chirothrips manicatus* Haliday and *Haplothrips aculeatus* (Fabricius), were found in all plant communities. The first was classified as a eudominant species in four plant communities (E-A, H-J, C-G, A), and the second one in two plant communities (C-A, E-S). *Ch. manicatus* had the highest share (93.2%) in the insect assemblage of the E-A community, with wingless individuals dominating. The other 22 species collected in this assemblage are recedents or subrecedents, eight of which are also graminicolous species. In the other plant communities, except for E-A, the higher share together with *Ch. manicatus* was exhibited by floricolous *Ceratothrips ericae* (Haliday), *Thrips physapus* Linnaeus, and *Haplothrips jasionis* Priesner in H-J; graminicolous *Anaphothrips obscurus* (Müller) and hygrophilous *A. badius* Williams in C-G; graminicolous *Aptinothrips stylifer* Trybom and floricolous *Frankliniella intonsa* (Trybom) in A. *H. aculeatus*, together with *F. intonsa*, was an eudominant in the C-A and E-S communities, accounting for 58.2% and 29.9% in the first and 36.2% and 47.3% in the latter, respectively. The following thrips species may be classified into the group of eudominats in the other studied plant communities: *Oxythrips bicolor* (O.M.Reuter), the most numerous species in the spring in L-P; *C. ericae* feeding on plants of the family *Ericaceae* mainly in E-P; *F. intonsa* in V-P and Mn; *A. rufus* (Haliday) in alder forest S-A and R-A, and the cosmopolitan *Thrips tabaci* Lindeman in A-C (Table 1 and Table S1).

**Table 1:** Checklist of species found in plant communities (quantitative study) of the Slowiński National Park.

Species	Code	L-P	E-P	E-A	H-J	V-P	S-A	R-A	V-B	C-G	C-A	E-S	Mn	A-C	A
*Aeolothrips albicinctus*	*Ae_al*	0.05	0.6			0.2	1.3		3.0			0.1			
*A. ericae*	*Ae_er*				0.3				0.2						
*A. fasciatus*	*Ae_fa*			0.1											0.4
*A. intermedius*	*Ae_in*														0.6
*A. melaleucus*	*Ae_me*						0.1		0.8						
*Anaphothrips badius*	*An_ba*						0.1	3.5	0.4	20.0	0.8				
*A. obscurus*	*An_ob*	3.6	1.1	0.1	0.1	0.9	14.3	12.9	13.2	33.2	0.8	0.2		13.8	3.0
*Aptinothrips rufus*	*Ap_ru*	5.8	7.6	0.9	1.0	2.9	49.1	38.8	19.4				12.8	5.5	13.6
*A. stylifer*	*Ap_st*	12.1	7.2	1.1	1.5	2.9	18.7	4.6	11.7			0.2	1.3	1.0	0.6
*Baliothrips dispar*	*Ba_di*							2.4							
*Bolacothrips jordani*	*Bo_jo*							2.4							0.4
*Ceratothrips ericae*	*Ce_er*	0.05	22.2	0.1	9.7	13.6			7.5	7.4	1.2	10.7			
*Chirothrips hamatus*	*Ch_ha*												1.3		
*C. manicatus*	*Ch_ma*	15.4	5.4	93.2	48.0	3.8	0.9	5.8	10.4	20.0	5.3	0.9	12.2	7.7	24.4
*C. pallidicornis*	*Ch_pa*														0.2
*Dendrothrips saltator*	*De_sa*	0.5						1.2							
*Euchaetothrips kroli*	*Eu_kr*							2.4				0.1			
*Frankliniella intonsa*	*Fr_in*	0.1	3.7	0.1	1.0	62.0			0.4	2.4	29.9	47.3	32.7	16.1	16.6
*F. tenuicornis*	*Fr_te*			0.1		0.2			0.2						
*Hemianaphothrips articulosus*	*He_ar*									2.4				0.4	1.0
*Iridothrips iridis*	*Ir_ir*							2.4							
*Limothrips cerealium*	*Li_ce*	1.0	0.2	0.4	0.1										0.2
*L. denticornis*	*Li_de*	0.6	0.6	0.5	0.3	1.6	1.2		1.5	2.4	1.6	0.2		0.4	0.4
*Mycterothrips latus*	*My_la*	0.1	0.2				0.4		0.6						
*M. salicis*	*My_sa*			0.1											
*Oxythrips ajugae*	*Ox_aj*	11.2	1.5	0.5	0.6	0.5	1.5	8.2	8.3			0.1			0.6
*O. bicolor*	*Ox_bi*	26.5	1.0	0.1		0.7	11.8	4.6	6.0		0.8	0.2	0.6	0.3	10.8
*Pelikanothrips kratochvili*	*Pe_kr*										0.4				
*Platythrips tunicatus*	*Pl_tu*												0.6		0.4
*Rhaphidothrips longistylosus*	*Rh_lo*												1.3		
*Rubiothrips ferrugineus*	*Ru_fe*	0.05												8.7	
*R. sordidus*	*Ru_va*													0.1	
*Scolothrips longicornis*	*Sc_lo*	0.05													
*Sericothrips bicornis*	*Se_bi*														0.4
*Taeniothrips picipes*	*Ta_pi*	10.1	10.8	0.1		2.9			6.0			0.2			
*T. zurstrassenii*	*Ta_zu*										0.8		0.6		
*Tenothrips frici*	*Te_fr*			0.1	0.4										
*Theilopedothrips pilosus*	*The_pi*														0.2
*Thrips alni*	*Th_al*	0.05													
*T. angusticeps*	*Th_an*								0.2					1.0	
*T. atratus*	*Th_at*	1.8	17.2	0.1		0.2						2.6	1.9	4.5	1.0
*T. discolor*	*Th_di*													0.4	4.0
*T. flavus*	*Th_fl*	2.5	9.3			0.5						0.2			
*T. fuscipennis*	*Th_fu*	1.0	0.1	0.1	0.3	0.2		2.4	1.8			0.1	1.9	10.0	1.8
*T. major*	*Th_mj*	0.9	2.9	0.1		0.5		3.6	0.2		0.8	0.1		0.4	1.0
*T. mancosetosus*	*Th_ma*												1.3	0.6	
*T. minutissimus*	*Th_mi*	1.9							3.8				2.6	0.6	0.2
*T. montanus*	*Th_mo*												12.2		
*T. nigropilosus*	*Th_ni*		0.2		0.1										0.6
*T. physapus*	*Th_ph*	0.05	0.2	0.1	10.8								4.2	1.0	4.2
*T. pini*	*Th_pi*	0.3													
*T. tabaci*	*Th_ta*	0.2	6.1	1.0	8.9	0.6	0.1	1.2	0.9			0.5	0.6	20.6	2.4
*T. trehernei*	*Th_tr*												1.3	1.0	0.4
*T. urticae*	*Th_ur*	0.2													1.8
*T. validus*	*Th_va*	0.1	0.1										5.1		1.8
*T. vulgatissimus*	*Th_vu*	0.1	0.1											4.3	
*Tmetothrips subapterus*	*Tm_su*	0.1								2.4				0.4	0.2
*Bolothrips dentipes*	*Bo_de*	0.1				0.2				2.4					
*B. icarus*	*Bo_ic*				0.1										
*Cephalothrips monilicornis*	*Ce_mo*	0.1	0.1	0.3	0.6	0.4			0.2						0.2
*Haplothrips aculeatus*	*Ha_ac*	2.4	1.6	0.7	0.3	5.2	0.3	2.4	1.8	7.4	58.2	36.2	4.2	0.6	1.6
*H. arenarius*	*Ha_ar*				0.4										
*H. distinguendus*	*Ha_di*			0.1					0.2					0.6	
*H. jasionis*	*Ha_ja*	0.1			15.4										
*H. leucanthemi*	*Ha_le*														4.8
*H. niger*	*Ha_ni*												1.3		
*H. phyllophilus*	*Ha_ph*	0.1					0.2		0.4						
*H. statices*	*Ha_st*				0.1										0.4
*H. subtilissimus*	*Ha_su*	0.1													
*Liothrips setinodis*	*Lio_se*	0.6													
*Xylaplothrips fuliginosus*	*Xy_fu*							1.2	0.9						
No. of species		36	24	23	21	20	14	17	26	10	10	17	19	24	33

**Figure 4:**
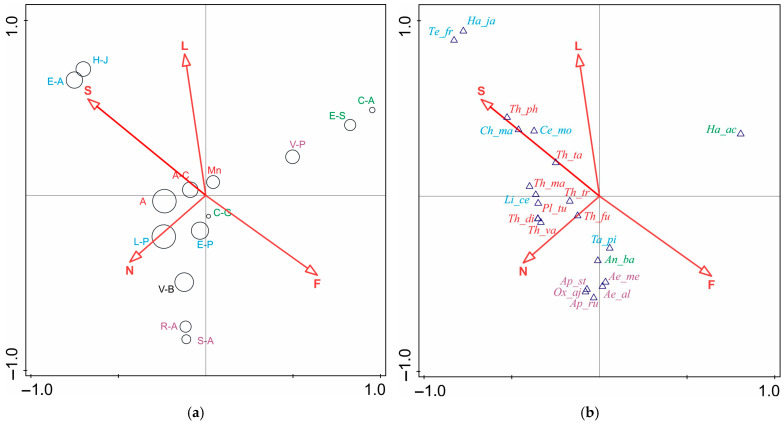
Ordination CCA biplots showing the impact of significant (*p* < 0.05) environmental parameters on Thysanoptera assemblages and the position of the species. (**a**)—CCA biplot of species assemblages by habitat variables, (**b**)—CCA biplot of thrips species by habitat variables. The diameter of the circles is correlated with the number of species in each assemblage. F—soil moisture, L—light intensity, N—general nutrient availability, S—soil salinity.

The acronyms used for the plant communities are explained in the descriptions of the plant communities and in the caption of Figure 1. The numbers indicate the dominance of individual species in the plant communities. Some of the codes of the thrips names are used in Figure 4b.

Of the 90 thrips species identified, monophagous insects constituted 17.8% (16 spp.), oligophagous—42.2% (38 spp.), and polyphagous—40.0% (36 spp.). Twenty-one species were associated with trees, including four fungivorous and six predatory species. The latter group included species of the genera *Aeolothrips* and *Scolothrips*. Graminicolous insects constituted 21% (19 spp.), with the remaining and the largest group of species—herbicolous (67.8% and 61 spp.) feeding on flowers or leaves of herbaceous dicotyledonous plants (Figure 2a, Table S1).

In terms of geographical distribution, 47.7% of all recorded species have a European range, and 18.9% have a Holarctic range. The other taxa have a much smaller share, ranging from 2.2% (Western Palearctic) with two species, *Taeniothrips picipes* (Zetterstedt) and *Scolothrips longicornis* (Priesner), to 10% (Palearctic) with nine species. The species classified as European and Asian were probably introduced from the west to the east, this group contains the foliophags, often harmful for trees or crops: *Dendrothrips saltator* Uzel, *Thrips minutissimus* Linnaeus, *T. nigropilosus* Uzel (Figure 2b, Table S1).

**Figure 3:**
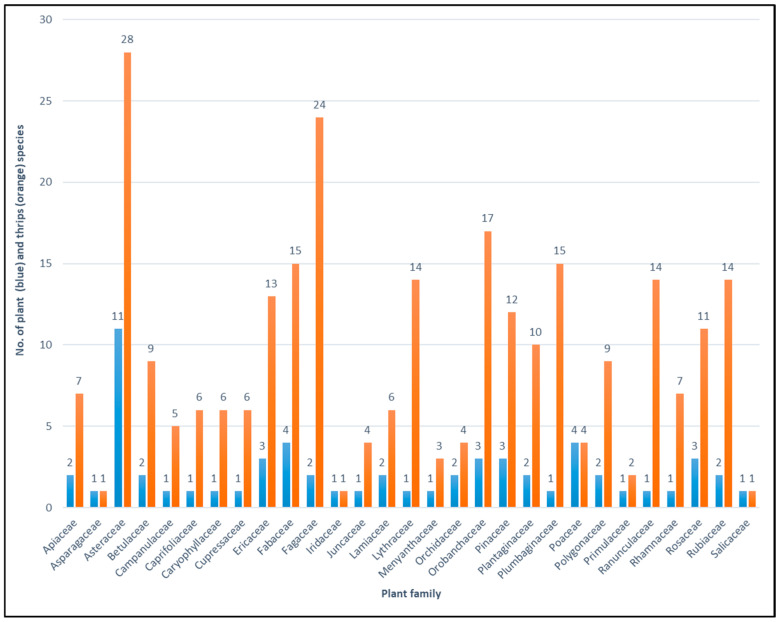
Comparison of the number of plants and thrips species related to the plants in particular plant families.

During the qualitative study, 61 plant species, both monocotyledonous and dicotyledonous, belonging to 29 families were analyzed. A total of 74 thrips species were collected from different parts of herbaceous plants or by shaking branches of shrubs and trees and tapping dead trunks lying on the forest floor. Using these methods, both adults and larvae were collected. The highest number of thrips species (28) was found on plants from the *Asteraceae* and the *Fagaceae* (24) families. Thirteen plant families were represented by only one species; three of these families: *Lythraceae*, *Plumbaginaceae*, and *Ranunculaceae* were the feeding sites of 14 or 15 thrips species. Conversely, the families *Asparagaceae*, *Salicaceae*, and *Iridaceae* were represented by one plant and one insect species. The latter family includes *Iris pseudacorus*, the host plant of *Iridothrips iridis* (Watson), which was identified in both the quantitative and qualitative studies. Searching for insects directly on plants allows collecting monophagous species together with their larvae, indicating a strong connection during feeding and breeding with those plants and enabling their identification as their hosts [28]. This group includes the following species: *Ctenothrips distinctus* (Uzel), *Rubiothrips silvarum* (Priesner), *Scolothrips uzeli* Schille, *Thrips dilatatus* Uzel, *Thrips juniperinus* Linnaeus, *Thrips menyanthidis* Bagnall, and *Thrips sambuci* Heeger, as well as four (*Megathrips lativentris* (Heeger), *Hoplothrips corticis* (De Geer), *Hoplothrips pedicularius* (Haliday) and *Phlaeothrips coriaceaus* Haliday.) mycophagous species associated with trunks of dead trees of the family *Fagaceae*. These species were noted individually (Figure 3, Table S2).

### 3.2. Relationships Between Thrips Assemblages and Environmental Factors

The similarity percentage analysis (SIMPER) of dominance showed that 19 thrips species were responsible for the differences between the four habitat types, i.e., costal dunes and forests (*cost*), bog forests (*swf*), peat bogs and marshes (*pbm*), and meadows (*med*), contributing 80% to the total dissimilarity. The structure of individual dominance of thrips within particular plant community allowed identification of species whose shares had a significant impact on the formation of insect assemblages (Table 2).

Among the six environmental parameters considered, only four: soil salinity (S), light intensity (L), general nutrient availability (N), and soil moisture (F) had a statistically significant effect on the diversity of the thrips assemblages. They are responsible for 14.5% (*p* = 0.008), 12.8% (*p* = 0.01), 12.0% (*p* = 0.012), and 9.2% (*p* = 0.022) of the total variation, respectively. These parameters explained 77.8% of the overall variation of thrips assemblages. Factors such as soil reaction (R) and temperature (T) did not significantly affect thrips occurrence in the assemblages across the different habitats. Soil salinity played the most significant role in shaping the thrips assemblages found in the coastal dunes (E-A, H-J). General nutrient availability influenced the development of thrips assemblages in the forests, whereas soil moisture was the additional parameter in the wet habitats (V-B, R-A, S-A). Light is a factor influencing the development of thrips assemblages in open, treeless habitats, in both dry (E-A, H-J) and more humid (C-A, E-S) environments (Figure 4a).

The results of the IndVal analysis indicated that the best candidates as indicator species for costal habitats were *Limothrips cerealium* Haliday, *Ch. manicatus*, *Cephalothrips monilicornis* (O.M.Reuter, *T. picipes*, *Tenothrips frici* (Uzel), and *H. jasionis*. The first three species are graminivorous, and the other three are floricolous, with *H. jasionis* strongly associated with the psammophilous plant *Jasione montana*, and *L. cerealium* occurring in large numbers in summer, especially before storms. For the swamp forests, the indicator species were the graminivores *Aptinothrips rufus* and *A. stylifer*, *Aeolothrips albicinctus* Haliday and *A*. *melaleucus* Haliday; the predators, which were more often caught from tree leaves; and *Oxythrips ajugae* (Uzel), which is particularly numerous in spring in the forests. *H. aculeatus* and *A. badius* are indicators for peat bogs and marshes. The former species is eurytopic and a standard component of thrips assemblages in grasslands and sedges, while the latter is a hygrophilous stenotope associated with peat bogs and fens. The species that are indicators of meadow communities are mostly floricolous eurytopes—*Thrips trehernei* Priesner, *T. fuscipennis* Haliday, *T. validus* Uzel, *T. tabaci*, and *T. physapus*. The exceptions include the monophagous *T. discolor* Haliday. and *T. mancosetosus* (Priesner), whose host plants are *Ranunculus repens* and *Cirsium oleraceum*, respectively, as well as *Platythrips tunicatus* (Haliday) feeding mainly on *Galium* spp. The selection of the above-mentioned species as distinguishing thrips groups was confirmed by their statistical significance (*p* < 0.005) (Figure 4b).

**Figure 5:**
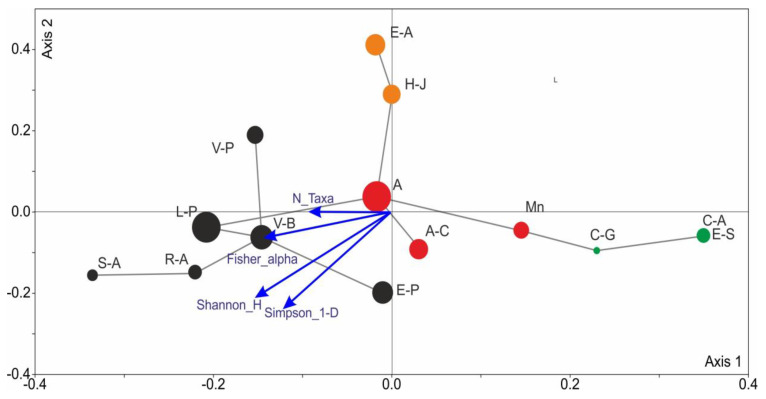
Two-dimensional non-metric multidimensional scaling (NMDS) plot of study sites representing different plant community types (red—meadows, green—peat bogs and marshes, orange—psammophilous grasslands, black—forests) vs. indices of biodiversity (SP—species richness, S-W —Shannon–Wiener diversity index, SIMP—Simpson dominance index, FISH—Fisher’s alpha) based on dissimilarities among thrips assemblages (Bray–Curtis distance matrix). Stress value = 0.105. The diameter of the circles is correlated with the number of species in each assemblage.

The non-metric multidimensional scaling (NMDS) method showed that the 14 thrips assemblages formed four groups that clearly differed in both species composition and abundance (Figure 5). Among them, E-A and H-J are characterized by similar numbers of species and a preference for soils with higher salinity and higher level of light. Communities occurring in humid habitats: peat bogs and marshes (C-G, C-A, E-S) are the most isolated in the graph due to the smallest number of species and the presence of hygrophilous species, such as *Anaphothrips badius* and *Pelikanothrips kratochvili* (Pelikán). These assemblages lacked *Aptinothrips rufus*, which was collected in abundance in all the other assemblages. On the opposite side of axis 1 were the thrips assemblages collected in the forest communities; among them were the alder forests (S-A, R-A) developing in wet habitats and characterized by the occurrence of hygrophilous species, such as *A. badius*, *Euchaetothrips kroli* (Schille), *I. iridis*, and *Baliothrips dispar* (Haliday). The other assemblages diagnosed in the coniferous forests were characterized by a higher abundance of arboricolous species of the genera *Oxythrips* and *Mycterothrips*, except for E-P, where *Ceratothrips ericae* and *Taeniothrips picipes* were caught in large numbers on *Ericaceae* plants dominating the undergrowth. Thysanoptera assemblages associated with the meadow plant communities were characterized by the occurrence of thrips species associated with flowering dicotyledonous plants, which affected their diversity and abundance (Figure 5).

It is worth noting that a species new to science, *Taeniothrips zurstrassenii* Zawirska, was discovered in the C-A and Mn plant communities, with *Cirsium palustris* as its host plant [54].

## 4. Discussion

Słowiński National Park is one of the 23 National Parks in Poland with unique moving dunes formed by wind-driven sand across or along the beach. Due to aeolian processes and wind activity over the course of one or more years, the landscape and vegetation change in this park [6]. In addition to natural phenomena, human activity has a significant impact on the current landscape and vegetation, which are threatened. Threats to dune habitats are primarily caused by technical shore protection, recreational pressure, and afforestation [55,56]. National parks are established to protect the most valuable natural assets, which in the SNP include dry grasslands and pine forests growing in the poor, sandy areas of shifting dunes. Meadows, peat bogs, and wet forests grow on acidic soils in the depressions beyond the dunes, surrounding the lakes found within this park. The majority of research sites constitute valuable and endangered habitats across the European continent. These habitats are listed in Annex 1 of the Habitats Directive [57], which means that they are legally protected within the European Union. For instance, *Elymo-Ammophiletum* and *Helichryso-Jasionetum* are classified as habitat 2130: fixed coastal dunes with herbaceous vegetation (‘grey dunes’). *Empetro nigri-Pinetum* is categorized as 2180: wooded dunes of the Atlantic, Continental, and Boreal region. *Vaccinio uliginosi-Pinetum* and *Vaccinio uliginosi-Betuletum* p*ubescentis* are classified as 91D0: bog woodland. Finally, *Erico-Sphagnetum medii* is classified as habitat 2190: humid dune slacks.

Our research was carried out in selected stations located in the most valuable areas, where 90 thrips species were found. This number constitutes approximately 40% of Thysanoptera recorded in Poland so far [29]. Among the species found, 80% (72 spp.) belonged to the suborder Terebrantia and 20% (18 spp.) to the suborder Tubulifera; species that feed on herbaceous plants predominated. The number of species with different food preferences depends on the methods used to collect thrips. In our research, we primarily used an entomological net and the method of shaking herbaceous plants and tree branches. These methods led to the identification of mainly floricolous and foliicolous thrips species on herbaceous plants. Only four species were identified as mycophagous. In contrast, in studies conducted across different microhabitats and ecosystems, e.g., in the Itanagar Wildlife Sanctuary, the Keibul Lamjao NP (India), and the Great Smoky Mountains National Park (USA), the primary methods were pitfall traps, including Malaise traps and Berlese funnels. In the aforementioned protected areas, spore-feeding tubuliferan species living under the bark and in leaf litter were collected in higher numbers. In the first area, the authors identified 108 thrips species, of which 24 were tubuliferans associated with dry twigs and litter. Among them, 17 species were spore- or hyphae-feeding [18]. Approximately 60% of the 119 species collected in another national park in India (the Keibul Lamjao NP) belonged to the suborder Tubulifera, and 46% were mycophagous [19]. In the studied American NP, 43 species were found, approximately half of which belonged to the suborder Tubulifera [21].

The knowledge on the assemblages of Thysanoptera in most of Polish national parks is limited. In addition to the results of thrips studies in the SNP presented here, studies on the diversity of the Thysanoptera fauna in various plant communities were conducted in five other national parks in Poland. A similar number of thrips species was recorded in Poleski NP (93 spp.), whereas smaller numbers were noted in Roztocze NP (77 spp.), Kampinoski NP (73 spp.), Ojców NP (71 spp.), and Babia Góra NP (53 spp.) [22,23,24,25,26,27]. Due to its boreal character, Poleski National Park is most similar to Słowiński National Park in terms of plant communities and the structure of thrips assemblages. The peat bog communities dominating this area are surrounded by forests such as *Vaccinio-uliginosi-Pinetum*, *Vaccinio uliginosi-Betuletum pubescentis*, and *Ribo-nigri-Alnetum*. Thrips assemblages noted in Poleski National Park were similar to those found in SPN, with a predominance of grass species: *Ch. manicatus*, *L. denticornis*, and *H. aculeatus*. Both parks also hosted hygrophilous species rare in Poland and Europe, such as *Pelikanothrips kratochvili*, *Thrips menyanthidis*, *Anaphothrips badius*, and *Bolothrips dentipes* [14,58,59]. These species, except for the last one, together with *Scolothrips uzeli*, *Mycterothrips albidicornis*, *Ctenothrips distinctus*, and *Haplothrips arenarius* are considered endangered and included in the Red List of Threatened Species in Central and Eastern Poland [59]. In addition, *T. menyanthidis* and *Iridothrips iridis* have been included in the List of Endangered Species in Germany [60]. Rozhina and Boklykova [12] conducted a study of thrips in the southern part of the Curonian Spit. They analyzed the fauna of raised bogs, pine forests, meadows, and dune complexes and identified 35 species from 18 genera. In their study, the most abundant species was *Thrips tabaci*, found on plants from 17 plant families. *Thrips tabaci* was also the most abundant species in research on the diversity of Thysanoptera in Southern France [61]. Of 53 thrips species collected from 108 plant species, *T. tabaci* was found on 99 species, followed by *Thrips major*, which was recorded on 54 plant species. In our study, *T. tabaci* was not abundant in most of the plant communities examined, owing to their natural character and the absence of anthropogenic disturbance. Only in the meadows of the *Angelico-Cirsietum* community was this species classified as eudominant. Among the thrips species recorded in the Curonian Spit habitats, the authors identified rare species also found in the SNP: *Bolothrips dentipes*, *H. arenarius*, and *A. badius*. In our study, we did not collect the leaf-feeding *Drepanothrips reuteri* Uzel or the floricolous *Thrips linarie* (Priesner), the latter feeding on and breeding on *Linaria vulgaris* Mill. Although it is a common plant species in Poland, *T. linarie* has not yet been recorded in the country [12,29].

It was interesting that, in our study, we did not find *Aptinothrips rufus* and *A. stylifer* (except for E-S) in peat bogs and marsh communities developing in deflation depressions, where temperature and humidity conditions are subject to greater fluctuations during the growing season than in other plant communities in the SNP [6]. On the other hand, these species were abundant in alder and birch forests developed in wet habitats, with fluctuations in the water level during the vegetative season. Additionally, studies conducted in peat bog communities in Poleski and Roztocze National Parks (eastern Poland) revealed that both *Aptinothrips* species were absent or collected singly [22,23,24]. In studies conducted in the mountains, differences in the occurrence of both *Aptinothrips* species were observed. *A. stylifer* was more resistant to unfavorable conditions—large temperature fluctuations and stronger winds—and was recorded seven times more frequently by Pelikan [62] in the higher zones of the Alps than *A. rufus*. Similar differences were observed in Babia Góra NP (West Carpathians), where *A. rufus* was less numerous in the highest vegetation zones [27]. These observations may indicate low tolerance of these species to the extreme conditions of wetland habitats and a stronger association with grasses prevalent in meadows and forests than with sedges dominating in peat bogs. Such food preferences were also confirmed by studies on the Curonian Spit, where both species were collected from grasses, with *A. stylifer* occurring in greater numbers [12].

These rare species, mentioned earlier, along with many others identified in the SNP, are primarily associated with wetlands, grasslands, forests, and coastal ecosystems, which are among the most threatened in Europe [57,63]. Particularly, species-poor plant communities, such as *Elymo-Ammophiletum* and *Helichryso-Jasionetum*, occurring on shifting dunes in the immediate vicinity of the sea are exposed to abiotic factors, such as strong winds prevailing from the sea towards the land [7]. Also the CCA analysis confirmed a positive correlation between thrips assemblage compositions with such factors as light and highly saline soil and a negative correlation with soil moisture. Grasses dominate the first community, while the second one, in addition to grasses, includes the psammophilous dicotyledonous plants *Helichrysum arenaria* and *Jasione montana*. In both communities, thrips assemblages were dominated by *Ch. manicatus*. Among the collected individuals of this species, wingless forms predominated, suggesting that they complete their entire development cycle in grass inflorescences and are carried over long distances by wind. Larvae, pupae, and adults of this species have a wedge-shaped, narrowed body, short antennae, and short legs; these features are excellent adaptations to life in the spikelets of *Leymus arenarius* (L.) Hochst., *Ammophila arenaria* (L.) Link, and *Corynephorus canescens* (L.) P. Beauv., which dominate among plant species [38,64]. *Haplothrips arenarius*, *H. statices*, and *H. jasionis* are monophagous, with *H. arenaria*, *Armeria elongata*, and *J. montana* as their respective hosts. The latter thrips species, apart from *Ch. manicatus*, was the most numerous in the insect assemblage occurring in the *Helichryso-Jasionetum* community. In the forests and grasslands on the dunes, *Limothrips cerealium* was noted. This graminicolous species is known as the Thunder Fly for its mass flight in the summer [30,38]. When the temperature rises to 20 degrees Celsius or higher, females synchronize their development and form large groups that fly over long distances on the wind and then land on a new host. Their males are wingless and die shortly after copulation. The phenomenon of mass thrips invasions is known for species such as *Limothrips denticornis*, *Ch. manicatus*, and *Taeniothrips* sp. The invasions can be irritating to humans and may damage crops, and they will intensify as temperatures rise due to global warming [30,38,65].

The pine forests predominate in the SNP; especially interesting and occurring exclusively on the coast is *Empetro nigri-Pinetum*, which is included on the list of Natura 2000 habitats. The thrips assemblage in this community is characterized by the highest abundance of *Ceratothrips ericae*, a eudominant species that feeds and breeds on plants of the fsmily *Ericaceae*. Inland, it occurs mainly on *Calluna vulgaris*, whereas in our study, both adults and larvae were collected on *Empetrum nigrum*, *Erica tetralix*, and *C. vulgaris*. Compared with the other two, the first species is closely associated with the poor, sandy substrate of coastal forests and peat bogs along the Baltic coast, which are listed in the Natura 2000 network. This network contains more habitats typical of the Baltic Sea shore, which are threatened by natural and human-caused environmental changes [6,55,63].

Climate change is a significant factor influencing the habitat’s surface area and structure. A more intensive survey of Odonata in Słowiński NP has revealed an increase in the number of thermophilic dragonfly species, which are expanding their geographical range and occurring beyond their natural habitats [10].

Rising temperatures and reduced precipitation can alter the local flora, which is crucial for herbivorous insects such as thrips (Thysanoptera). Polyphagous and oligophagous species may find new plants for feeding and breeding; in turn, monophagous species may lose their hosts, which could disappear when the habitat dries or when the synchrony between the species life cycle and their host development is disturbed [38,66,67,68]. In our study, we observed that a relatively small number of thrips species with broad food and habitat preferences dominated in terms of abundance and were classified as eudominants or dominants at most sites. In contrast, stenotopic species with narrow food preferences, although represented by numerous taxa, were rare in particular thrips assemblages and may be most sensitive to environmental changes. In Europe, some exotic thrips species known from greenhouses so far, i.e., *Microcephalothrips abdominalis* (Crawford), *Hercinothrips femoralis* (Reuter), and *Frankliniella occidentalis* (Pergande), have expanded their range and are observed in natural habitats. In the context of global climate change, they may exhibit high invasion potential [61,69,70,71].

## 5. Conclusions

Our research, conducted across 14 diverse plant communities in Słowiński National Park, identified 90 Thysanoptera species representing approximately 40% of the fauna in Poland. This number included a similar share of species found using both qualitative and quantitative methods, which complemented each other. Therefore, in terms of species diversity and thrips assemblages, the SPN is one of the best-studied areas in the country. The importance of the SNP in preserving the biological diversity of thrips is evidenced by the discovery of many rare, both in Poland and in Europe, species as well as a species new to science (*Taeniothrips zurstrassenii* Zaw.), known only from Poland so far. The analysis of environmental factors, including soil salinity, moisture content, nutrient availability, and light intensity, revealed their significant impact on thrips assemblage diversity. The IndVal analysis identified thrips species within individual assemblages, grouping them according to the habitat. Anthropogenic changes and natural processes occurring in the environment have the greatest impact on plant communities and associated insect groups on shifting dunes.

## Figures and Tables

**Table 2:** Taxon-specific dissimilarities in four habitat types—similarity percentage analysis (SIMPER). Av. dissim.—average dissimilarity, Contrib. %—percentage contribution, Cumulative %—cumulative percentage contribution, Mean—mean dissimilarity.

Taxon	Av. Dissim.	Contrib. %	Cumulative %	Mean *cost*	Mean *swf*	Mean *pbm*	Mean *med*
*Chirothrips manicatus*	7.3	13.2	13.2	40.5	5.2	8.7	14.8
*Frankliniella intonsa*	7.1	12.8	26.0	1.2	15.6	26.2	21.8
*Aptinothrips rufus*	5.2	9.5	35.4	3.8	27.5	0.0	10.6
*Haplothrips aculeatus*	5.0	9.0	44.5	1.3	2.4	33.9	2.1
*Anaphothrips obscurus*	3.1	5.7	50.2	1.2	10.3	11.4	5.6
*Ceratothrips ericae*	2.3	4.1	59.4	8.0	5.3	6.5	0.0
*Oxythrips bicolor*	2.1	3.8	63.2	6.9	5.8	0.3	3.9
*Aptinothrips stylifer*	2.0	3.6	66.8	5.5	9.5	0.1	1.0
*Thrips tabaci*	1.6	2.8	69.6	4.1	0.7	0.2	7.9
*Taeniothrips picipes*	1.1	2.0	71.7	5.3	2.2	0.1	0.0
*Oxythrips ajugae*	1.1	2.0	73.7	3.5	4.6	0.0	0.2
*Anaphothrips badius*	1.1	2.0	75.7	0.0	1.0	6.9	0.0
*Thrips atratus*	1.1	2.0	77.7	4.8	0.1	0.9	2.5
*Thrips physapus*	0.8	1.5	79.2	2.8	0.0	0.0	3.1
*Thrips fuscipennis*	0.7	1.3	81.9	0.4	1.1	0.0	4.6
*Haplothrips jasionis*	0.7	1.2	83.1	3.9	0.0	0.0	0.0
*Thrips montanus*	0.6	1.0	86.4	0.0	0.0	0.0	4.1
*Thrips flavus*	0.5	0.9	88.3	3.0	0.1	0.1	0.0
*Rubiothrips ferrugineus*	0.4	0.7	89.9	0.0	0.0	0.0	2.9

Codes of habitat types: *cost*—costal dunes and forests, *swf*—bog forests, *pbm*—peat bogs and marshes, and *med*—meadows.

## Data Availability

The original contributions presented in this study are included in the article/Supplementary Materials. Further inquiries can be directed to the corresponding author.

## Supplementary Materials

The following supporting information can be downloaded at: https://www.mdpi.com/article/10.3390/insects17010119/s1, Table S1: The distribution and ecology characteristics of all thrips species collected in the Słowiński National Park. Table S2: Checklist of plant species, plant families, and thrips species collected on particular plants (qualitative studies) in the Slowiński National Park.
