# Supplementary material for: Diversity and Ecology of Thrips (Thysanoptera, Insecta) Assemblages in Słowiński National Park—A Biosphere Reserve on the Baltic Coast (Northern Poland)
Source: Insects. 2026 Jan 21;17(1):119. doi: 10.3390/insects17010119 (PMC12842015; doi:10.3390/insects17010119)
Supplement: Supplementary file 1 [file insects-17-00119-s001.zip › Table S1. Checklist_distribution-ecology.pdf]

Kucharczyk, H.; Kucharczyk, M; Zawirska, I.†. Diversity and Ecology of Thrips (Thysanoptera, Insecta) Assemblages in Słowiński National Park—A Biosphere Reserve on the Baltic Coast (Northern Poland)

Table S1. The distribution and ecology characteristics of thrips species collected in the Słowinski National Park

	**Thrips species**	**Distribution**	**Ecology**
1.	*Aeolothrips albicinctus* Haliday	HOL	gr, pol
2.	*Aeolothrips ericae* Bagnall	HOL	fl, ol
3.	*Aeolothrips fasciatus* (Linnaeus)	HOL	zoo, pol, fl
4.	*Aeolothrips intermedius* Bagnall	PAL	zoo, pol, fl
5.	*Aeolothrips melaleucus* Haliday	HOL	zoo, ol, ar
6.	*Aeolothrips versicolor* Uzel	HOL	fol, ol, ar
7.	*Anaphothrips badius* Williams	EUR	fol, ol, hig
8.	*Anaphothrips obscurus* (Müller)	COS	gr, ol
9.	*Aptinothrips rufus* (Haliday)	COS	gr, pol
10.	*Aptinothrips stylifer* Trybom	HOL	gr, pol
11.	*Baliothrips dispar* (Haliday)	EUS	fol, pol, hig
12.	*Bolacothrips jordani* Uzel	EUR	gr, pol
13.	*Ctenothrips distinctus* (Uzel)	EUR	fol, mon
14.	*Ceratothrips ericae* (Haliday)	HOL	fl, ol
15.	*Chirothrips hamatus* Trybom	HOL	gr, pol, hig
16.	*Chirothrips manicatus* Haliday	HOL	gr, pol
17.	*Chirothrips pallidicornis* (Priesner)	EUR	gr, pol
18.	*Dendrothrips degeeri* Uzel	EUR	fol, ol, ar
19.	*Dendrothrips saltatrix* Uzel	EUR+ASIA	fol, pol, ar
20.	*Euchaetothrips kroli* (Schille)	EUR	gr, ol, hig
21.	*Frankliniella intonsa* (Trybom)	COS	fl, pol
22.	*Frankliniella tenuicornis* (Uzel)	COS	gr, pol
23.	*Hemianaphothrips articulosus* Priesner	EUR	gr, ol, hig
24.	*Iridothrips iridis* (Watson)	EUR	fol, mon, hig
25.	*Limothrips cerealium* Haliday	COS	gr, pol
26.	*Limothrips denticornis* Haliday	HOL	gr, pol
27.	*Mycterothrips albidicornis* (Knechtel)	EUR	fol, ol, ar
28.	*Mycterothrips consociatus* (Targioni-Tozetti)	PAL	fol, ol, ar
29.	*Mycterothrips latus* Bagnall	EUS	fol, ol, ar
30.	*Mycterothrips salicis* (O.M.Reuter)	EUS	fol, ol, ar
31.	*Neohydatothrips gracilicornis* (Williams)	PAL	gr, ol
32.	*Oxythrips ajugae* (Uzel)	EUR	fol, ol, ar
33.	*Oxythrips bicolor* (O.M. Reuter)	EUR	fol, ol, ar
34.	*Pelikanothrips kratochvili* (Pelikán)	EUR	fol, ol, hig
35.	*Pezothrips frontalis* (Uzel)	EUR	fol, ol, xer
36.	*Platythrips tunicatus* (Haliday)	EUR	fl, ol
37.	*Rhaphidothrips longistylosus* Uzel	HOL	gr, pol
38.	*Rubiothrips ferrugineus* (Uzel)	EUR	fl, ol
39.	*Rubiothrips silvarum* (Priesner)	EUS	fl, mon, xer
40.	*Rubiothrips sordidus* (Uzel)	EUS	fl, ol
41.	*Scolothrips longicornis* (Priesner)	HOL	fol, pol, zoo
42.	*Scolothrips uzeli* Schille	EUR	fl, mon, zoo
43.	*Sericothrips bicornis* (Karny)	EUR	fl, ol
44.	*Taeniothrips picipes* (Zetterstedt)	W-PAL	fl, pol
45.	*Taeniothrips zurstrasseni* Zawirska	EUR	fl, mon
46.	*Tenothrips frici* (Uzel)	PAL	fl, pol
47.	*Theilopedothrips pilosus* (Uzel)	EUR	fl, ol, xer

48.	*Thrips alni* Uzel	EUR	fol, mon, ar
49.	*Thrips angusticeps* Uzel	W-PAL	fl, pol
50.	*Thrips atratus* (Haliday)	HOL	fl, pol
51.	*Thrips difficilis* Priesner	EUR	fol, ol, ar
52.	*Thrips dilatatus* Uzel	EUS	fl, ol
53.	*Thrips discolor* Haliday	EUR	fl, mon, hig
54.	*Thrips flavus* Schrank	COS	fl, pol
55.	*Thrips fuscipennis* Haliday	PAL	fl, fol, pol
56.	*Thrips juniperinus* Linnaeus	EUR	fl, mon, ar
57.	*Thrips major* Uzel	PAL	fl, fol,pol
58.	*Thrips mancosetosus* (Priesner)	EUR	fl, mon, hig
59.	*Thrips menyanthidis* Bagnall	EUR	fol, mon, hig
60.	*Thrips minutissimus* Linnaeus	EUR+ASIA	fol, ol, ar
61.	*Thrips montanus* (Priesner)	EUR	fl, mon
62.	*Thrips nigropilosus* Uzel	EUR+ASIA	fl, fol, pol
63.	*Thrips physapus* Linnaeus	EUS	fl, pol
64.	*Thrips pilichi* Priesner	EUR	fl, ol
65.	*Thrips pini* (Uzel)	HOL	fl, fol, ol, ar
66.	*Thrips sambuci* Heeger	EUR	fol, mon, ar
67.	*Thrips tabaci* Lindeman	COC	fl, fol, pol
68.	*Thrips trehernei* Priesner	HOL	fl, pol
69.	*Thrips urticae* Fabricius	EUR	fl, mon
70.	*Thrips validus* Uzel	EUR+ASIA	fl, pol
71.	*Thrips vulgatissimus* (Haliday)	EUR	fl, ol
72.	*Tmetothrips subapterus* (Haliday)	EUR	fol, ol, hig
73.	*Bolothrips dentipes* (O.M.Reuter)	EUR	gr, ol, hig
74.	*Bolothrips icarus* (Uzel)	EUR	gr, ol, xer
75.	*Cephalothrips monilicornis* (O.M. Reuter)	HOL	gr, ol
76.	*Haplothrips aculeatus* (Fabricius)	PAL	gr, pol
77.	*Haplothrips arenarius* Priesner	EUR	fl, mon, xer
78.	*Haplothrips distinguendus* (Uzel)	EUR+ASIA	fl, fol, pol
79.	*Haplothrips jasionis* Priesner	EUR	fl, mon, xer
80.	*Haplothrips leucanthemi* (Schrank)	HOL	fl, mon
81.	*Haplothrips niger* (Osborn)	PAL	fl, ol
82.	*Haplothrips phyllophilus* Priesner	EUR	fol, pol, ar, cor
83.	*Haplothrips statices* (Haliday)	EUR	fl, mon, xer
84.	*Haplothrips subtilissimus* (Haliday)	PAL	fol, pol, cor, ar
85.	*Hoplothrips corticis* (De Geer)	HOL	mf, ol, ar, cor
86.	*Hoplothrips pedicularius* (Haliday)	EUR	mf, ol. ar, cor
87.	*Liothrips setinodis* (O.M. Reuter)	EUR	fol, ol, ar
88.	*Megathrips lativentris* (Heeger)	EUR	mf, ol, ar, cor
89.	*Phlaeothrips coriaceus* Haliday	EUR	mf, ol, ar, cor
90.	*Xylaplothrips fuliginosus* (Schille)	EUR	zoo, ar, cor

Abbreviations: **distribution** – COS – cosmopolite, EUR – European, EUR+ASIA – European and Asian, EUS – Eurosiberian, HOL – Holarctic, PAL – Palearctic, W-PAL – West-Palearctic; **ecology** – mo – monophagous, ol – oligophagous, pol – polyphagous, fl – floricolous, fol – foliicolous, gr – graminicolous, zoo – zoophagous, ar – arboricolous, hig – higrophilous, xer - termophilous, cor – corticolous, mf – mycophagous, ar – arboricolous
