# Supplementary material for: Diversity and Ecology of Thrips (Thysanoptera, Insecta) Assemblages in Słowiński National Park—A Biosphere Reserve on the Baltic Coast (Northern Poland)
Source: Insects. 2026 Jan 21;17(1):119. doi: 10.3390/insects17010119 (PMC12842015; doi:10.3390/insects17010119)
Supplement: Supplementary file 1 [file insects-17-00119-s001.zip › Table S2. Checklist of plant species.pdf]

Kucharczyk, H.; Kucharczyk, M; Zawirska, I.✝. Diversity and Ecology of Thrips (Thysanoptera, Insecta) Assemblages in Słowiński National Park—A Biosphere Reserve on the Baltic Coast (Northern Poland)

Table S2. Checklist of plant families, plant species, and thrips species collected on particular plants (qualitative studies) in the Słowiński National Park

Plant family	Plant species	Thysanoptera species
Apiaceae	*Anthriscus sylvestris*	*Haplothrips aculeatus*<br>*Limothrips denticornis*<br>*Thrips fuscipennis*<br>*Thrips minutissimus*<br>*Thrips physapus*<br>*Thrips tabaci*<br>*Thrips vulgatissimus*
Apiaceae	*Daucus carota*	*Thrips fuscipennis*<br>*Thrips tabaci*
Asparagaceae	*Convallaria majalis*	*Ctenothrips distinctus*
Asteraceae	*Achillea millefolium*	*Thrips tabaci*
Asteraceae	*Cirsium arvense*	*Ceratothrips ericae*<br>*Chirothrips manicatus*<br>*Frankliniella intonsa*<br>*Haplothrips aculeatus*<br>*Haplothrips jasionis*<br>*Mycterothrips consociatus*<br>*Oxythrips ajugae*<br>*Thrips atratus*<br>*Thrips flavus*<br>*Thrips fuscipennis*<br>*Thrips major*<br>*Thrips physapus*<br>*Thrips tabaci*<br>*Thrips vulgatissimus*
Asteraceae	*Cirsium oleraceum*	*Apitnothrips rufus*<br>*Frankliniella intonsa*<br>*Haplothrips aculeatus*<br>*Thrips atratus*<br>*Thrips flavus*<br>*Thrips fuscipennis*<br>*Thrips major*<br>*Thrips mancosetosus*<br>*Thrips physapus*<br>*Thrips tabaci*<br>*Thrips validus*<br>*Thrips vulgatissimus*
Asteraceae	*Cirsium palustre*	*Chirothrips manicatus*<br>*Ceratothrips ericae*<br>*Euchaetothrips kroli*<br>*Frankliniella intonsa*<br>*Limothrips consimilis*<br>*Haplothrips aculeatus*<br>*Haplothrips distinguendus*<br>*Thrips atratus*<br>*Thrips flavus*<br>*Thrips fuscipennis*<br>*Thrips major*<br>*Thrips mancosetosus*<br>*Thrips physapus*<br>*Thrips tabaci*<br>*Tmetothrips subapterus*<br>*Liothrips setinodis*
Asteraceae	*Cirsium vulgare*	*Frankliniella intonsa*<br>*Thrips physapus*
Asteraceae	*Crepis paludosa*	*Thrips validus*
Asteraceae	*Helichrysum arenarium*	*Chirothrips manicatus*<br>*Haplothrips arenarius*

		Haplothrips jasionis<br>*Thrips fuscipennis*<br>*Thrips major*<br>*Thrips physapus*<br>*Thrips tabaci*
Asteraceae	*Hieracium umbellatum*	*Chirothrips manicatus*<br>*Frankliniella intonsa*<br>*Haplothrips aculeatus*<br>*Haplothrips arenarius*<br>*Haplothrips jasionis*<br>*Tenothrips frici*<br>*Thrips difficilis*<br>*Thrips major*<br>*Thrips physapus*<br>*Thrips tabaci*<br>*Thrips trehernei*<br>*Thrips validus*
Asteraceae	*Leontodon autumnalis*	*Frankliniella intonsa*<br>*Thrips physapus*<br>*Thrips tabaci*<br>*Thrips trehernei*<br>*Thrips validus*
Asteraceae	*Leucanthemum vulgare*	*Aeolothrips intermedius*<br>*Chirothrips manicatus*<br>*Frankliniella intonsa*<br>*Haplothrips aculeatus*<br>*Haplothrips leucanthemi*<br>*Thrips atratus*<br>*Thrips flavus*<br>*Thrips fuscipennis*<br>*Thrips major*<br>*Thrips physapus*<br>*Thrips pilichi*<br>*Thrips tabaci*<br>*Thrips vulgatissimus*
Asteraceae	*Sonchus arvensis*	*Frankliniella intonsa*<br>*Thrips atratus*<br>*Thrips trehernei*
Betulaceae	*Betula pendula*	*Mycterothrips consociatus*
Betulaceae	*Betula verrucosa*	*Aeolothrips melaleucus*<br>*Chirothrips manicatus*<br>*Limothrips denticornis*<br>*Mycterothrips consociatus*<br>*Oxythrips ajugae*<br>*Oxythrips bicolor*<br>*Taeniothrips picipes*<br>*Thrips alni*<br>*Thrips minutissimus*<br>*Thrips tabaci*
Campanulaceae	*Jasione montana*	*Chirothrips manicatus*<br>*Frankliniella intonsa*<br>*Haplothrips arenarius*<br>*Thrips physapus*<br>*Thrips tabaci*
Caprifoliaceae	*Succisa pratensis*	*Frankliniella intonsa*<br>*Haplothrips aculeatus*<br>*Thrips major*<br>*Thrips mancosetosus*<br>*Thrips montanus*<br>*Thrips tabaci*
Caryophyllaceae	*Stellaria media*	*Anaphothrips obscurus*<br>*Frankliniella intonsa*<br>*Thrips atratus*<br>*Thrips fuscipennis*<br>*Thrips tabaci*<br>*Thrips validus*
Cupressaceae	*Juniperus communis*	*Oxythrips ajugae*<br>*Oxythrips bicolor*<br>*Thrips juniperinus*<br>*Thrips minutissimus*

		Anaphothrips obscurus<br>*Scolothrips uzeli*
Ericaceae	*Calluna vulgaris*	*Anaphothrips obscurus*<br>*Aptinothrips rufus*<br>*Ceratothrips ericae*<br>*Chirothrips manicatus*<br>*Frankliniella intonsa*<br>*Taeniothrips picipes*<br>*Thrips major*<br>*Thrips atratus*<br>*Thrips flavus*<br>*Thrips fuscipennis*<br>*Thrips nigropilosus*<br>*Thrips tabaci*<br>*Cephalothrips monilicornis*
Ericaceae	*Empetrum nigrum*	*Ceratothrips ericae*
Ericaceae	*Erica tetralix*	*Ceratothrips ericae*<br>*Chirothrips manicatus*<br>*Frankliniella intonsa*<br>*Haplothrips aculeatus*<br>*Taeniothrips picipes*<br>*Thrips flavus*<br>*Thrips fuscipennis*<br>*Thrips major*<br>*Thrips physapus*<br>*Thrips tabaci*
Fabaceae	*Cytisus scoparius*	*Aeolothrips ericae*<br>*Aeolothrips intermedius*<br>*Frankliniella intonsa*<br>*Thrips angusticeps*<br>*Thrips atratus*<br>*Thrips flavus*<br>*Thrips fuscipennis*<br>*Thrips major*<br>*Thrips physapus*<br>*Thrips tabaci*<br>*Thrips trehernei*<br>*Thrips vulgatissimus*
Fabaceae	*Lathyrus japonicus*	*Chirothrips manicatus*<br>*Thrips atratus*<br>*Thrips physapus*<br>*Thrips tabaci*
Fabaceae	*Lotus corniculatus*	*Frankliniella intonsa*
Fabaceae	*Vicia tetrasperma*	*Aeolothrips fasciatus*<br>*Apitnothrips rufus*<br>*Chirothrips manicatus*<br>*Frankliniella intonsa*<br>*Thrips fuscipennis*<br>*Thrips major*<br>*Thrips physapus*<br>*Thrips tabaci*
Fagaceae	*Fagus sylvatica*	*Aeolothrips versicolor*<br>*Aptinothrips rufus*<br>*Dendrothrips degeeri*<br>*Dendrothrips saltatrix*<br>*Haplothrips aculeatus*<br>*Haplothrips phyllophilus*<br>*Hoplothrips pedicularius*<br>*Liothrips setinodis*<br>*Oxythrips ajugae*<br>*Oxythrips bicolor*<br>*Thrips minutissimus*
Fagaceae	*Quercus robour*	*Aeolothrips melaleucus*<br>*Aeolothrips versicolor*<br>*Anaphothrips obscurus*<br>*Chirothrips manicatus*<br>*Haplothrips aculeatus*<br>*Haplothrips phyllophilus*<br>*Hoplothrips corticis*<br>*Liothrips setinodis*

		Megathrips lativentris<br>*Mycterothrips albidicornis*<br>*Oxythrips ajugae*<br>*Oxythrips bicolor*<br>*Phlaeothrips coriaceus*<br>*Taeniothrips picipes*<br>*Thrips alni*<br>*Thrips fuscipennis*<br>*Thrips major*<br>*Thrips minutissimus*<br>*Thrips pini*<br>*Xylaplothrips fuliginosus*
Iridaceae	*Iris pseudacorus*	*Iridothrips iridis*
Juncaceae	*Juncus effusus*	*Aptinothrips rufus*<br>*Chirothrips manicatus*<br>*Oxythrips bicolor*<br>*Iridothrips iridis*
Lamiaceae	*Stachys palustris*	*Frankliniella intonsa*<br>*Haplothrips aculeatus*<br>*Taeniothrips zurstrasseni*<br>*Thrips fuscipennis*<br>*Thrips major*
Lamiaceae	*Galeopsis tetrahit*	*Chirothrips manicatus*<br>*Frankliniella intonsa*<br>*Thrips fuscipennis*
Lythraceae	*Lythrum salicaria*	*Aeolothrips fasciatus*<br>*Aeolothrips intermedius*<br>*Apitnothrips rufus*<br>*Aptinothrips stylifer*<br>*Frankliniella intonsa*<br>*Haplothrips aculeatus*<br>*Mycterothrips consociatus*<br>*Taeniothrips zurstrasseni*<br>*Thrips atratus*<br>*Thrips fuscipennis*<br>*Thrips physapus*<br>*Thrips trehernei*<br>*Thrips major*<br>*Thrips tabaci*
Menyanthaceae	*Menyanthes trifoliata*	*Anaphothrips obscurus*<br>*Thrips fuscipennis*<br>*Thrips menyanthidis*
Orchidaceae	*Epipactis atrorubens*	*Aptinothrips rufus*<br>*Haplothrips aculeatus*<br>*Thrips tabaci*
Orchidaceae	*Goodyera repens*	*Thrips validus*
Orobanchaceae	*Euphrasia stricta*	*Aeolothrips fasciatus*<br>*Aeolothrips intermedius*<br>*Frankliniella intonsa*<br>*Thrips dilatatus*<br>*Thrips mancosetosus*
Orobanchaceae	*Melampyrum pratense*	*Aeolothrips intermedius*<br>*Aeolothrips ericae*<br>*Aptinothrips rufus*<br>*Aptinothrips stylifer*<br>*Ceratothrips ericae*<br>*Frankliniella intonsa*<br>*Haplothrips aculeatus*<br>*Mycterothrips latus*<br>*Taeniothrips picipes*<br>*Thrips flavus*<br>*Thrips major*<br>*Thrips tabaci*
Orobanchaceae	*Rhinanthus serotinus*	*Aeolothrips fasciatus*<br>*Aeolothrips intermedius*<br>*Aptinothrips stylifer*<br>*Frankliniella intonsa*<br>*Haplothrips aculeatus*<br>*Thrips atratus*<br>*Thrips dilatatus*<br>*Thrips flavus*

		Thrips fuscipennis<br>*Thrips major*<br>*Thrips montanus*<br>*Thrips tabaci*
Pinaceae	*Larix europaea*	*Aeolothrips melaleucus*<br>*Aptinothrips stylifer*<br>*Chirothrips manicatus*<br>*Limothrips cerealium*<br>*Mycterothrips consociatus*
Pinaceae	*Picea alba*	*Oxythrips ajugae*<br>*Oxythrips bicolor*<br>*Thrips minutissimus*<br>*Liothrips setinodis*<br>*Mycterothrips consociatus*<br>*Thrips pini*
Pinaceae	*Pinus sylvestris*	*Chirothrips manicatus*<br>*Ceratothrips ericae*<br>*Oxythrips ajugae*<br>*Oxythrips bicolor*<br>*Thrips minutissimus*<br>*Thrips tabaci*
Plantaginaceae	*Veronica chamaedrys*	*Aeolothrips intermedius*<br>*Apitnothrips rufus*<br>*Aptinothrips stylifer*<br>*Frankliniella intonsa*<br>*Haplothrips distinguendus*<br>*Haplothrips leucanthemi*<br>*Pezothrips frontalis*<br>*Theilopedothrips pilosus*<br>*Thrips nigropilosus*
Plantaginaceae	*Linaria vulgaris*	*Frankliniella intonsa*<br>*Thrips tabaci*
Plumbaginaceae	*Armeria maritima*	*Aeolothrips intermedius*<br>*Apitnothrips rufus*<br>*Aptinothrips stylifer*<br>*Frankliniella intonsa*<br>*Haplothrips aculeatus*<br>*Haplothrips distinguendus*<br>*Haplothrips statices*<br>*Limothrips cerealium*<br>*Thrips atratus*<br>*Thrips flavus*<br>*Thrips fuscipennis*<br>*Thrips major*<br>*Thrips physapus*<br>*Thrips tabaci*<br>*Thrips vulgatissimus*
Poaceae	*Ammophila arenaria*	*Euchaetotchrips kroli*<br>*Hemianaphothrips articulosus*
Poaceae	*xCalammophila baltica*	*Hemianaphothrips articulosus*<br>*Chirothrips manicatus*
Poaceae	*Glyceria maxima*	*Euchaetothrips kroli*<br>*Hemianaphothrips articulosus*
Poaceae	*Phragmites australis*	*Chirothrips manicatus*<br>*Haplothrips aculeatus*
Polygonaceae	*Polygonum bistorta*	*Aeolothrips intermedius*<br>*Frankliniella intonsa*<br>*Haplothrips aculeatus*<br>*Thrips atratus*<br>*Thrips fuscipennis*<br>*Thrips mancosetosus*<br>*Thrips tabaci*<br>*Thrips validus*<br>*Thrips vulgatissimus*
Polygonaceae	*Rumex acetosa*	*Frankliniella intonsa*<br>*Haplothrips aculeatus*<br>*Thrips fuscipennis*<br>*Thrips tabaci*<br>*Thrips vulgatissimus*
Primulaceae	*Lysimachia vulgaris*	*Frankliniella intonsa*

		Thrips fuscipennis
Ranunculaceae	*Ranunculus acris i Ranunculus repens*	*Apitnothrips rufus*<br>*Chirothrips manicatus*<br>*Frankliniella intonsa*<br>*Oxythrips bicolor*<br>*Thrips angusticeps*<br>*Thrips discolor*<br>*Thrips fuscipennis*<br>*Thrips major*<br>*Thrips minutissimus*<br>*Thrips physapus*<br>*Thrips tabaci*<br>*Thrips trehernei*<br>*Thrips validus*<br>*Thrips vulgatissimus*
Rhamnaceae	*Frangula alnus*	*Aptinothrips stylifer*<br>*Haplothrips aculeatus*<br>*Haplothrips phyllophilus*<br>*Mycterothrips consociatus*<br>*Thrips fuscipennis*<br>*Thrips major*<br>*Thrips sambuci*
Rosaceae	*Geum rivale*	*Anaphothrips obscurus*<br>*Chirothrips manicatus*<br>*Frankliniella intonsa*<br>*Thrips fuscipennis*
Rosaceae	*Padus avium*	*Haplothrips phyllophilus*<br>*Oxythrips ajugae*<br>*Oxythrips bicolor*<br>*Thrips minutissimus*
Rosaceae	*Sorbus aucuparia*	*Haplothrips phyllophilus*<br>*Oxythrips ajugae*<br>*Oxythrips bicolor*<br>*Thrips minutissimus*<br>*Thrips major*<br>*Thrips sambuci*<br>*Thrips tabaci*
Rubiaceae	*Galium mollugo*	*Aeolothrips intermedius*<br>*Anaphothrips obscurus*<br>*Apitnothrips rufus*<br>*Chirothrips manicatus*<br>*Frankliniella intonsa*<br>*Haplothrips aculeatus*<br>*Mycterothrips salicis*<br>*Platythrips tunicatus*<br>*Rubiothrips silvarum*<br>*Rubiothrips sordidus*<br>*Thrips fuscipennis*<br>*Thrips major*<br>*Thrips tabaci*
Rubiaceae	*Galium palustre*	*Anaphothrips obscurus*<br>*Aptinothrips stylifer*<br>*Euchaetothrips kroli*
Salicaceae	*Salix sp.*	*Mycterothrips salicis*
